# Evaluation of the Pseudostatic Analyses of Earth Dams Using FE Simulation and Observed Earthquake-Induced Deformations: Case Studies of Upper San Fernando and Kitayama Dams

**DOI:** 10.1155/2014/585462

**Published:** 2014-01-27

**Authors:** Tohid Akhlaghi, Ali Nikkar

**Affiliations:** Faculty of Civil Engineering, University of Tabriz, Tabriz 5166116471, Iran

## Abstract

Evaluation of the accuracy of the pseudostatic approach is governed by the accuracy with which the simple pseudostatic inertial forces represent the complex dynamic inertial forces that actually exist in an earthquake. In this study, the Upper San Fernando and Kitayama earth dams, which have been designed using the pseudostatic approach and damaged during the 1971 San Fernando and 1995 Kobe earthquakes, were investigated and analyzed. The finite element models of the dams were prepared based on the detailed available data and results of in situ and laboratory material tests. Dynamic analyses were conducted to simulate the earthquake-induced deformations of the dams using the computer program Plaxis code. Then the pseudostatic seismic coefficient used in the design and analyses of the dams were compared with the seismic coefficients obtained from dynamic analyses of the simulated model as well as the other available proposed pseudostatic correlations. Based on the comparisons made, the accuracy and reliability of the pseudostatic seismic coefficients are evaluated and discussed.

## 1. Introduction

The seismic stability of earth structures has been analyzed by pseudostatic procedures for many decades in which the effects of an earthquake are represented by constant horizontal and/or vertical accelerations. Stability is expressed in terms of a pseudostatic factor of safety calculated by limit equilibrium procedures. Limit equilibrium analyses consider force and/or moment equilibrium of a mass of soil above a potential failure surface. The first explicit application of the pseudostatic approach to the analysis of seismic slope stability has been attributed to Terzaghi [1]. In their most common form, pseudostatic analyses represent the effects of earthquake shaking by pseudostatic accelerations that produce inertial forces which act through the centroid of the failure mass.

The results of pseudostatic analyses are critically dependent on the value of the seismic coefficient. Selection of an appropriate pseudostatic coefficient (particularly *k*_*h*_) is the most important, and the most difficult, aspect of a pseudostatic analysis. The seismic coefficient controls the pseudostatic force on the failure mass, so its value should be related to some measures of the amplitude of the inertial force induced in the potentially unstable material. If the slope material was rigid, the inertial force induced on a potential slide would be equal to the product of the actual horizontal acceleration and the mass of the unstable material. This inertial force would reach its maximum value when the horizontal acceleration reached its maximum value. In recognition of the fact that actual slopes are not rigid and that the peak acceleration exists for only a very short time, the pseudostatic coefficients used in practice generally correspond to acceleration values well below the maximum value. Terzaghi [1] originally suggested the use of *k*_*h*_ = 0.1 for sever earthquakes (Rossi-Forel IX), *k*_*h*_ = 0.2 for violent and destructive earthquakes (Rossi-Forel X), and *k*_*h*_ = 0.5 for catastrophic earthquakes. Seed [2] listed pseudostatic design criteria for 14 dams in 10 seismically active countries and 12 required minimum factors of safety of 1.0 to 1.5 with pseudostatic coefficients of 0.10 to 0.12. Marcuson [3] suggested that appropriate pseudostatic coefficients for dams should correspond to one-third to one-half of the maximum acceleration, including amplification or deamplification effects, to which the dam is subjected. Using shear beams models, Seed and Martin [4] and Dakoulas and Gazetas [5] showed that the inertial force on a potentially unstable slope in an earth dam depends on the response of the dam and that the average seismic coefficient for a deep failure surface is substantially smaller than that of a failure surface that does not extend far below the crest. Seed [2] also indicated that deformations of earth dams constructed of ductile soils with crest accelerations less than 0.75 g would be acceptably small for pseudostatic factors of safety of at least 1.15 with *k*_*h*_ = 0.1  (*M* = 6.5) to *k*_*h*_ = 0.15  (*M* = 8.25). This criteria would allow the use of pseudostatic accelerations as small as 13 to 20 percent of the peak crest acceleration. Hynes-Griffin and Franklin [6] applied the Newmark sliding block analysis to over 350 accelerograms and concluded that earth dams with pseudostatic factors of safety greater than 1.0 using *k*_*h*_ = 0.5*a*_max⁡_/*g* would not develop dangerously large deformations.

As can be seen from above discussions, there are no hard and fast rules for selection of a pseudostatic coefficient for design. However, it seems that the pseudostatic coefficient should be based on the actual anticipated level of acceleration in the failure mass and that it should correspond to some fractions of the anticipated peak acceleration, although engineering judgment is required for all cases.

Representation of the complex, transient, dynamic effects of earthquake shaking by a single constant unidirectional pseudostatic acceleration is obviously quite crude. Detailed analyses of historical and recent earthquake-induced landslides have illustrated significant shortcomings of the pseudostatic approach. Results of pseudostatic analyses of some earth dams (e.g., Upper San Fernando dam, Lower San Fernando dam, Sheffield dam, and Tailing dam) show that pseudostatic analyses produced factor of safety well above 1.0 for a number of dams that later failed during earthquakes. Romo and Seed [7] collected many of the destructed dams since 1900 to 1980 which had been designed using pseudostatic method. These are summarized and listed in Table 1. These cases illustrate the inability of the pseudostatic method to reliably evaluate the stability of slopes susceptible to weakening instability. Nevertheless, the pseudostatic approach can provide at least a crude index of relative, if not absolute, stability.

Despite the above-mentioned limitations, the pseudostatic approach has a number of attractive features. The analysis is relatively simple and straightforward. Indeed, its similarity to the static limit equilibrium analyses routinely conducted by geotechnical engineers makes its computations easy to understand and perform. It produces a scalar index of stability (the factor of safety) that is analogous to that produced by static stability analyses. It must always be recognized, however, that the accuracy of the pseudostatic approach is governed by the accuracy with which the simple pseudostatic inertial forces represent the complex dynamic inertial forces that actually exist in an earthquake. Difficulty in the assignment of appropriate pseudostatic coefficients and in interpretation of pseudostatic factors of safety, coupled with the development of more realistic methods of analysis, has reduced the use of the pseudostatic approach for seismic slope stability analyses. Methods based on evaluation of permanent slope deformation are being used increasingly for seismic slope stability analysis.

## 2. Case Studies

### 2.1. Upper San Fernando Dam

#### 2.1.1. Upper San Fernando Dam Geometry and Performance

The Upper San Fernando dam located northwest of Los Angeles and north of the Lower San Fernando dam was built in 1922 using semihydraulic fill technique [8]. The dam was about 24.4 meters high and was constructed upon 15.5 m of alluvial deposits overlying bedrock.

The 1971 San Fernando earthquake had a moment magnitude of 6.7 and an epicenter about 13 km from the dam site. The peak horizontal acceleration at the dam site was estimated to be around 0.6 g. Several longitudinal cracks were observed along almost the full length of the dam on the upstream slope slightly below the preearthquake reservoir level. The crest of the dam settled 0.76 m down and moved 1.5 m downstream. The maximum amount of horizontal displacements was about 2 m. Sand boils below the toe and increased water levels in three standpipe piezometers suggested that soil liquefaction had occurred. Water overflowed from two of piezometers. The reservoir level at the time of earthquake was at elevation 369.51 m, 1.83 m below the crest of the dam.

#### 2.1.2. Material Properties of Upper San Fernando Dam

**Figure 1:**
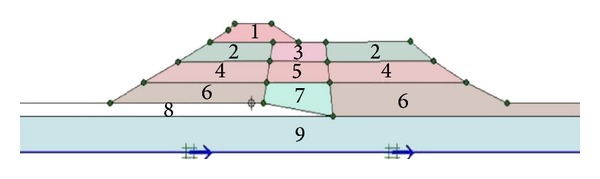
Cross section and layers of Upper San Fernando dam.

As shown in Figure 1, the dam is divided into 9 different layers, each representing a different soil material zone. These soil units of dam section have been classified using (1) suggested by Sawada and Takahashi [9]. The equation indicates the effect of height variation on the shear wave velocity and shear modulus of the layers materials located at different levels of the dam section. Consider

(1) $$\begin{array}{c} V_{S}=140Z^{0.34}, \end{array}$$

where *V*_*S*_ is the shear wave velocity and *Z* is the dam height below the crest.

The relationship between shear wave velocity, shear, and Young's module (*G* and *E*) is as follows:

(2) $$\begin{array}{c} G=\rho \times {V_{S}}^{2},\\ G=\frac{E}{2\left(1+\nu \right)}, \end{array}$$

where *ν* is the Poisson ratio.

The layers' soil parameters associated with the nine soil zones obtained using the above equations and detailed results given by Seed et al. [8] are listed in Table 2.

### 2.2. Kitayama Dam

#### 2.2.1. Kitayama Dam Geometry and Performance

The Kitayama dam is located on the Rokko granite zone and about 1.5 km from the Ashiya fault and the Koyo fault. The dam is an earth dam with a height of 25 meters and was completed in 1968.

**Figure 2:**
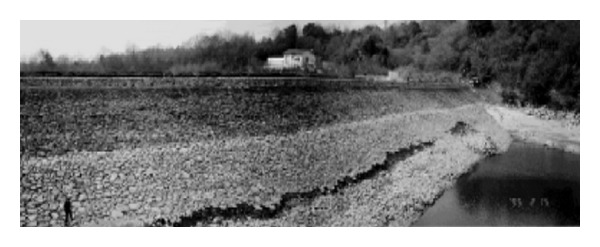
The sliding failure zone in Kitayama dam caused by Kobe 1995 earthquake.

The Kobe earthquake of January 17, 1995, in Japan caused slides on the upstream slope of the Kitayama dam which is an earth dam located about 33 kilometers northeast of the epicenter. It was minor damage that did not affect the structural safety and the water storage functions of the dam. But this was the first time that an embankment dam designed based on design standards and filled by the engineered rolling compaction was damaged in this way in Japan. The sliding failure zone with a depth of 1.5 to 2 m was confirmed and the length in the dam axis direction of the sliding was about 100 m (Figure 2).

The Kobe University motion is the earthquake motion observed during the Kobe earthquake. The observation station is located on a weathered rock with an S-wave velocity of 340 m/s and 24 km far from the epicenter. The Kobe University motion has a peak acceleration of 270.4 gal and duration of 20 sec. During the earthquake, the reservoir water level was at the top of the sliding failure block. The level differences on the top of the sliding block were from 1 to 1.5 m.

#### 2.2.2. Material Properties of Kitayama Dam

**Figure 3:**
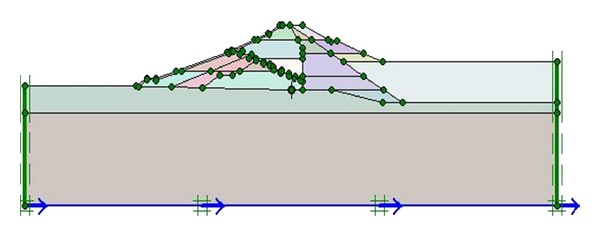
Cross section, layers, and stress points of Kitayama dam.

As shown in Figure 3, the dam is divided into 24 different layers, each representing a different soil material zone. These soil units of dam section have been also classified using (1). The layers' material properties associated with the 24 soil zones determined using the mentioned equations and detailed results given by Sakamoto et al. [10] are listed in Table 3.

## 3. Study Methodology

Most engineers consider the seismic coefficient as a means of designating the magnitude of a static force which is equivalent in effects (i.e., produces the same deformations of the earth dam) to the actual dynamic inertia forces induced by the earthquake. But how would the seismic coefficient denoting this equivalent static force be determined? It would seem that the determination of an appropriate value would necessarily involve two steps:

1. determination and specification of deformations and degree of instability of dam induced by the earthquake;
2. evaluation of equivalent static force with the capability to make the same displacements or instabilities.

It would appear that any attempt to select a final value of such a seismic coefficient without going through step (1) and without a large backlog of experience to guide the selection could have little reliable basis.

**Figure 4:**
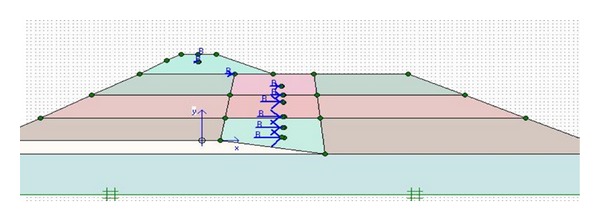
Equivalent static forces acting at layers gravity center.

In order to determine exact results for stage (1), it will be preferable to utilize dynamic analyses based on finite element method, and hence the Plaxis software seems to be an appropriate choice. High accuracy of dynamic analysis puts it at high point of view. The results obtained from two-dimensional dynamic analyses of dams under corresponding earthquake, such as horizontal and vertical displacements, almost justify the observed displacements. Then an equivalent static force is determined for each layer and seismic coefficient is obtained for those layers. In order to reach this aim, the static forces were activated to each layer's gravity center (as shown in Figure 4) and displacements and dam deformations were gained. The importance of this study shines in evaluating the varying seismic coefficient for dams and that is relevant to differentiation of each layer's coefficient of the dam. Assuming a constant seismic coefficient would be applicable for rigid structures and using this current method for earth dams which have not rigid-body response is not rational. Destruction of Lower San Fernando dam and Oshima Tailing dam confirms the invalidity of pseudostatic analysis with constant coefficient, since both of them had been designed using pseudostatic analysis having seismic coefficients of 0.15 and 0.2, respectively. In this study, the equivalent seismic coefficients for different soil zones of Upper San Fernando and Kitayama dams have been determined using two-dimensional dynamic analyses and large backlog, and then the results have been compared with the design seismic coefficient (0.15) of the dams.

## 4. Dynamic Analyses

Most of the problems encountered in the area of geotechnical engineering such as retaining walls, tunnels, earth dams, and embankments are studied using two-dimensional dynamic analyses based on the finite element method (FEM) which is one of the available powerful numerical methods. The fundamental stages required to create a FE model include selecting an appropriate element, dividing the model into elements and nods, extending equations of each element and determining element's stiffness matrix, combining element's matrix, and creating a single matrix for model. Elements movement equation is given by

(3) $$\begin{array}{c} \left[M\right]\left\{\overset{..}{U}\right\}+\left[C\right]\left\{\overset{.}{U}\right\}+\left[K\right]\left\{U\right\}=\left\{R\left(t\right)\right\}, \end{array}$$

in which [*M*] is the whole mass matrix, [*C*] is the whole damping matrix, [*U*] is the model nods axial movement, and {*R*(*t*)} is the axial force of model points.

One of the current methods used to solve the movement equation is the Newmark step-by-step method. Newmark [11] provided this method for dynamic analysis of earthquake loading. In this method, displacement and velocity are determined using the following equations:

(4) $$\begin{array}{c} u_{t+\Delta t}=u_{t}+{\overset{.}{u}}_{t}\Delta t+\left[\left(\frac{1}{2}-\alpha \right)\overset{..}{u}+\alpha {\overset{..}{u}}_{t+\Delta t}\right]\Delta t^{2},\\ {\overset{.}{u}}_{t+\Delta t}={\overset{.}{u}}_{t}+\left[\left(1-\beta \right){\overset{..}{u}}_{t}+\beta {\overset{..}{u}}_{t+\Delta t}\right]\Delta t, \end{array}$$

where Δ*t* is time pace and *α* and *β* are controlling parameters for numerical integration accuracy, according to the implicit the Newmark scheme [12]. In order to obtain a stable solution, these parameters have to satisfy the following condition:

(5) $$\begin{array}{c} \beta \geq 0.5;\;\alpha \geq 0.25{\left(0.5+\beta \right)}^{2}. \end{array}$$

In the classical Lagrange method in [13], *β* = 0.5 leads the calculations to rational results. Despite Newmark's damping method, taking advantage of *β* = 0.6 and *α* = 0.3025 values, in this study, average acceleration method is being used to solve movement equations, as well as Newmark's method.

Special boundary conditions have to be defined in order to avoid the spurious reflections of the waves on the model boundaries. These boundaries are based on the Lysmer-Kohlmeyer model. According to this model, the normal and shear stress components absorbed by a damper are determined as follows:

(6) $$\begin{array}{c} \sigma_{n}=-c_{1}\rho V_{P}{\dot{u}}_{x},\\ \tau =-c_{2}\rho V_{S}{\dot{u}}_{y}, \end{array}$$

where *ρ* is the mass density, *V*_*S*_ is the shear wave velocity, *V*_*P*_ are the longitudinal wave velocity, ${\dot{u}}_{x}$ and ${\dot{u}}_{y}$ = velocity of particle motion in the direction of *x* and *y*, respectively, and *c*_1_ and *c*_2_ are relaxation coefficients used to improve the wave absorption on the absorbent boundaries. *c*_1_ corrects the dissipation in the direction normal to the boundary and *c*_2_ in the tangential direction. The research and experience findings recommend to choose *c*_1_ = 1 and *c*_2_ = 0.25 for the best results [14].

## 5. Dam Simulation

**Figure 5:**
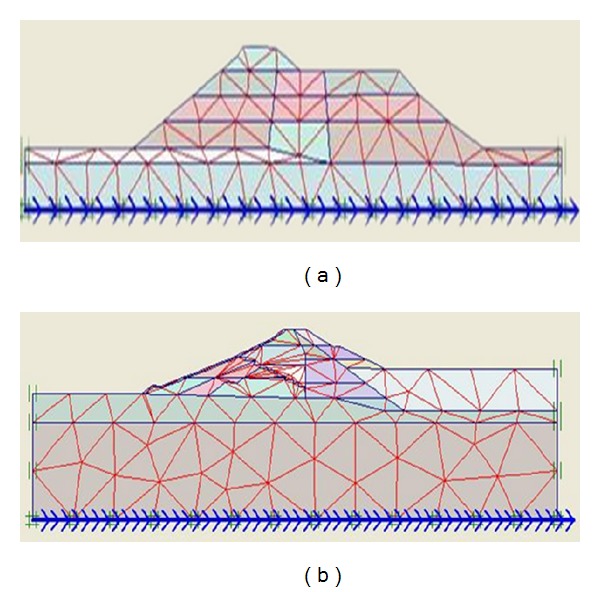
Section of generated mesh of (a) Upper San Fernando and (b) Kitayama dams.

**Figure 6:**
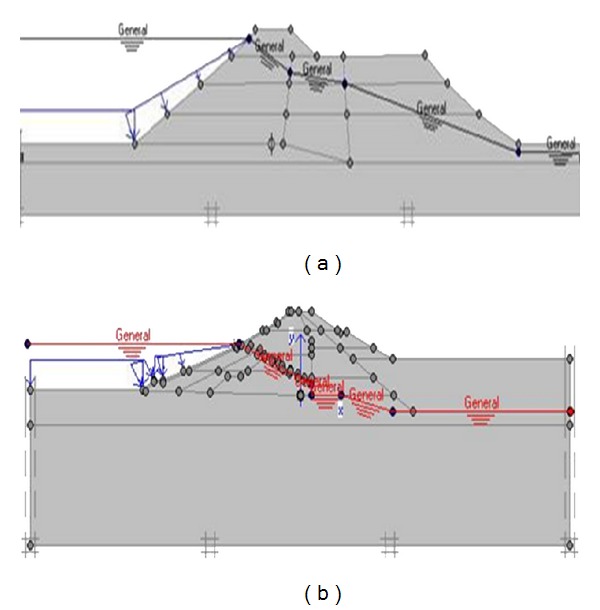
Upstream water level and phreatic line of (a) Upper San Fernando and (b) Kitayama dams.

The process begins with specifying the clusters (Upper San Fernando dam involved 9 clusters and Kitayama dam involved 24 clusters) and defining the properties relevant to each cluster.  Figure 5 shows the generated mesh sections and Figure 6 exhibits upstream water level and phreatic line of the dams.

**Figure 7:**
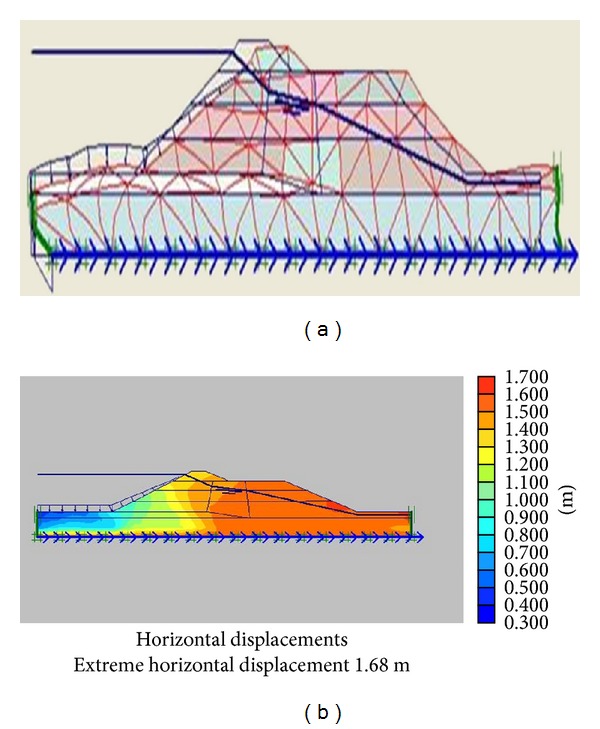
Whole deformations and horizontal displacements of Upper San Fernando dam.

**Figure 8:**
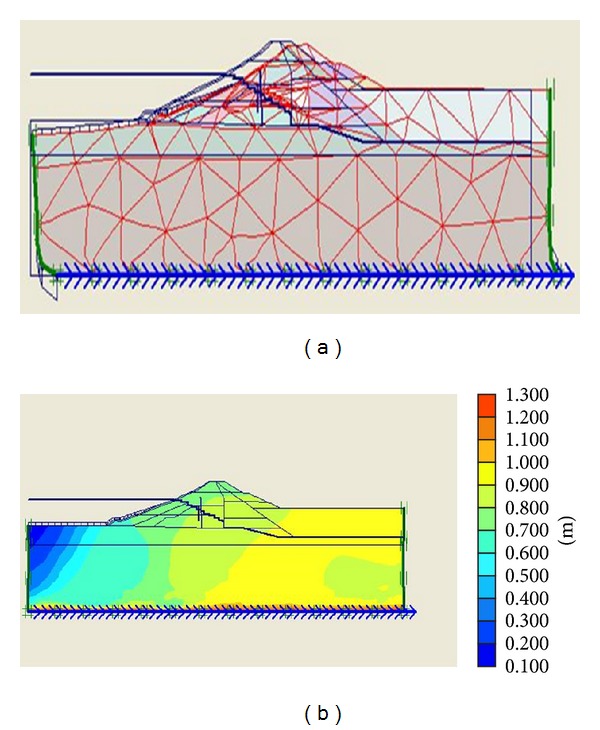
Whole deformations and horizontal displacements of Kitayama dam.

The numerical calculations using Plaxis software involve 3 phases. First phase is dam plastic analysis conducted for the time when the construction is over. Second phase includes dam plastic analyses under own body load and finally the last step consists of dynamic analysis under earthquake loading. The third phase loading is applied in the form of a file (accelerogram) input to the program. The whole deformations and horizontal and vertical displacements of the dams are obtained in the output of the program, but considering the importance of horizontal displacements, and for the sake of space saving, only the whole deformations and horizontal displacement diagrams are illustrated in Figures 7 and 8.

## 6. Stress-Time Analyses

**Figure 9:**
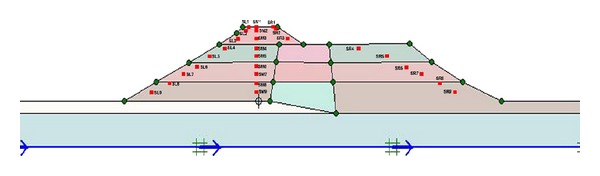
Location of stress points for Upper San Fernando dam.

**Figure 10:**
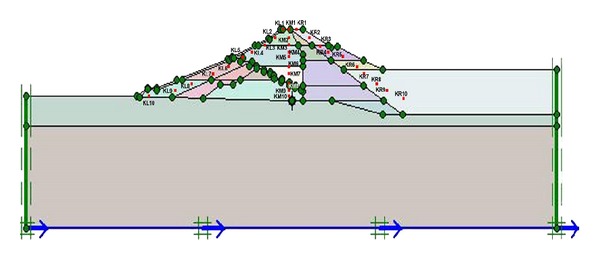
Location of stress points for Kitayama dam.

Before starting calculation step, stress points are chosen on cross section of the dam. These points are located in the *x* direction with three points at each level as shown in Figures 9 and 10 (K for Kitayama and S for San Fernando), one point upstream side (having symbol L), one point middle part (with symbol M), and one point downstream side (with symbol R). 27 points constituting 9 lines and 30 points constituting 10 lines parallel to the *x* direction are, respectively, specified for Upper San Fernando (represented by S) dam and Kitayama (represented by K) dam.

**Figure 11:**
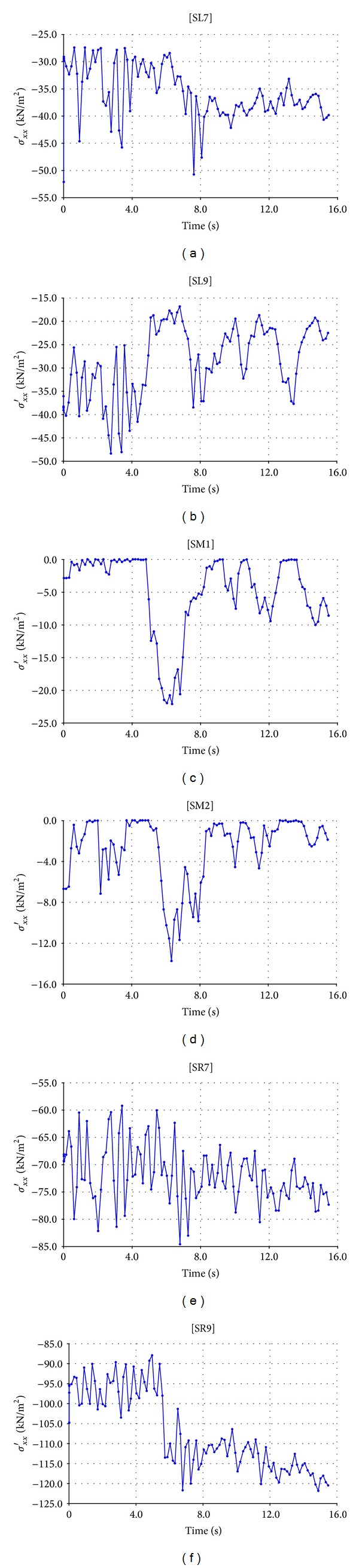
Stress-time curves for Upper San Fernando dam.

**Figure 12:**
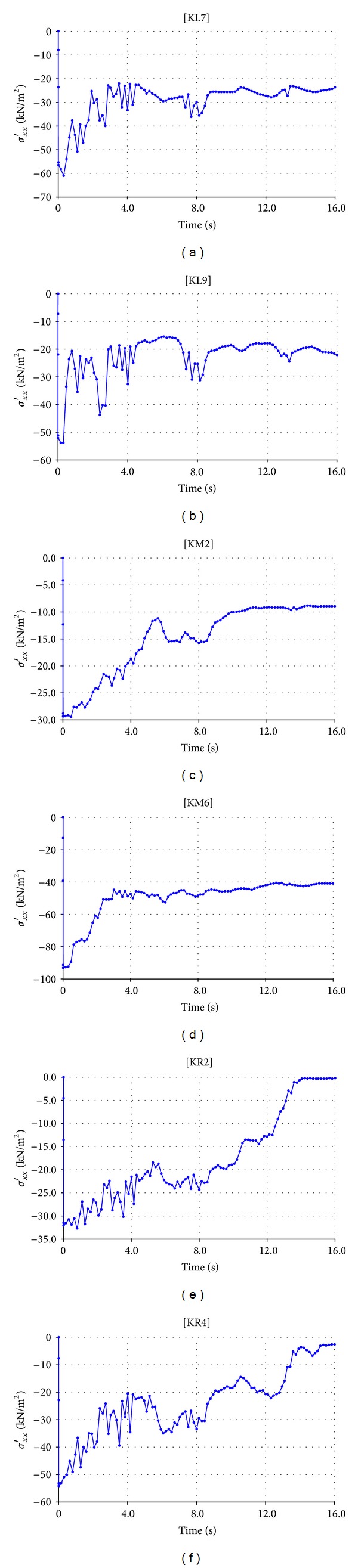
Stress-time curves for Kitayama dam.

Stress (*σ*_*xx*_)-time curves (27 curves for Upper San Fernando and 30 curves for Kitayama) can be obtained from CURVE step of the program. Owing to the generation of numerous curves and again for the sake of space saving, only some stress-time curves related to points at various levels of the sections are provided. Figures 11 and 12, respectively, show these envisaged curves for Upper San Fernando and Kitayama dams.

## 7. Determination of Equivalent Force

**Figure 13:**
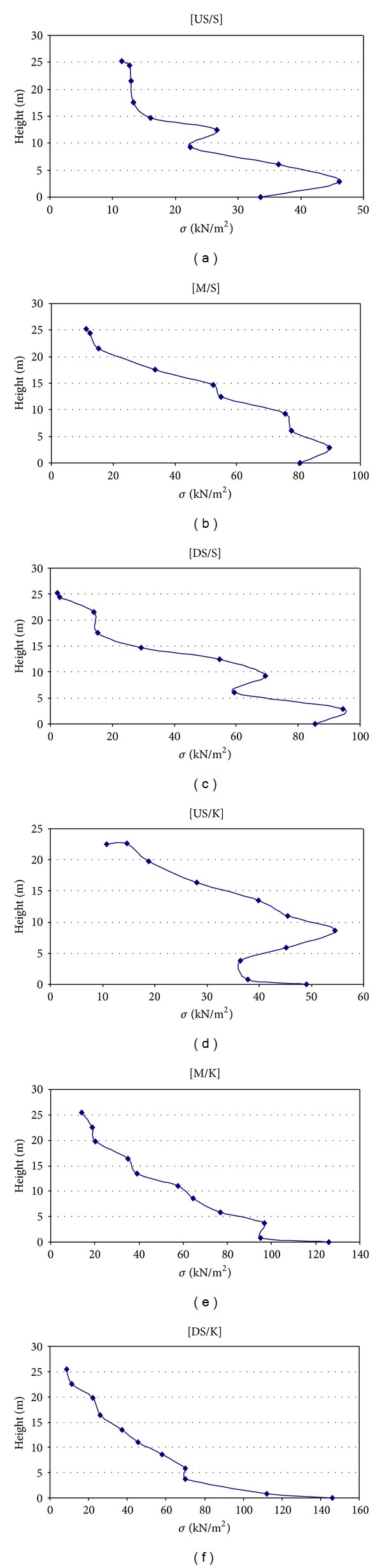
Approximated stress curves for various parts of Upper San Fernando (S) and Kitayama (K) dams.

For each curve, a maximum value is deliberated for a period and is considered due to its conservative value. Though the maximum value of each curve is multiplied by 0.7, the distribution of stress along the height of model is approximated to be linear for all points located in the upstream (US), downstream (DS), and middle (M) parts. The results are shown in Figure 13.

**Figure 14:**
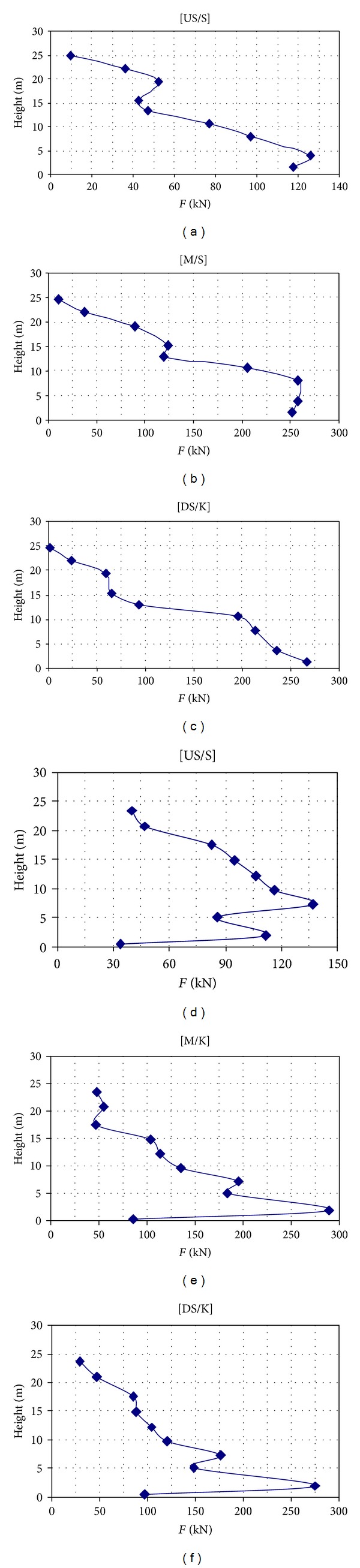
Equivalent force curves for various parts of Upper San Fernando (S) and Kitayama (K) dams.

The equivalent force for each part of layer across stress point is determined using the product of layer height to approximate stress value. The results are illustrated in Figure 14.

Then each layer's equivalent force can be determined by employing the following equation:

(7) $$\begin{array}{c} F=\overset{3}{\underset{i=1}{\sum }}\frac{F_{i}L_{i}}{L}, \end{array}$$

in which *F*_*i*_ is force of the part (upstream, middle, and downstream), *L*_*i*_ is the effective length of the part, and *L* is the layer's length. Finally seismic coefficient is calculated by dividing layer force to its weight. The results are summarized and listed in Table 4.

## 8. Conclusion

The results obtained from the analyses conducted for investigating the Upper San Fernando and Kitayama dams behavior, respectively, under San Fernando 1971 earthquake and Kobe 1995 earthquake loading indicate that the seismic coefficient increases with the increasing of the height. The ratio of seismic coefficient at the crest of the Upper San Fernando dam over seismic coefficient at the base of the dam is about 1.44 and this ratio for the Kitayama dam is about 2. For both dams the design seismic coefficient was 0.15, but the minimum calculated seismic coefficient for lower layers of the dams is 0.21. In Upper San Fernando case, the crest of the dam settled to 0.76 m and moved 1.5 m downstream and the maximum amount of horizontal displacements was about 2 m. In Kitayama case, the crest of the dam settled to 0.43 m and moved 0.72 m downstream and the maximum amount of horizontal displacements reached almost 0.98 m. The results indicate that the constant seismic coefficients used in designing both dams were not applicable and in case of using constant seismic coefficient it must be between the minimum value of 0.21 and the maximum value of 0.311 for Upper San Fernando case and between the minimum value of 0.21 and the maximum value of 0.47 for Kitayama case. The following results are gained by comparing the design seismic coefficient to the calculated seismic coefficients separately for both dams.

(*1) Upper San Fernando Dam*

- The maximum value of seismic coefficient offered by USBR is 0.2, while this coefficient is not applicable in the case of this dam.
- The minimum acceleration submitted by seismograph has a value about 0.34 g but considering the rigid-body response method (*k* = 0.34) in this case which has a maximum seismic coefficient of 0.31 is extremely conservative.
- The maximum and minimum values determined using Ambraseys' method [15] are 0.33 and 0.23, respectively. It seems that the rational value for seismic coefficient exists between these evaluated values.

(*2) Kitayama Dam*

- The maximum value of seismic coefficient offered by JCOLD is 0.25 which is not applicable in the case of this dam too.
- Although the minimum acceleration recorded in the seismograph has a value about 0.221 g but considering the rigid-body response method (*k* = 0.221), which has a maximum seismic coefficient of 0.47, is not reliable.
- In this case, the maximum and minimum values determined using Ambraseys' method are 0.27 and 0.23, respectively. As mentioned above, the rational value for seismic coefficient exists between these computed values.

Finally this study shows that considering inconstant seismic coefficient in earth dam design is more realistic and rational than considering a constant seismic coefficient. Furthermore the procedure employed in this study can be utilized for evaluation of design seismic coefficient of constructed earth dams designed using pseudostatic analyses.

## Figures and Tables

**Table 1:** Examples of destructed dams since 1900 to 1980 [7].

	Number of embankments		
Earthquake location	Year	Magnitude	Damages
In USA			
San Francisco	1906	8.2	5 dams
Kern County	1952	7.6	3 dams
Fallon	1954	6.7	2 dams
San Fernando	1971	6.6	2 dam
Santa Barbara	1925	6.3	1 dam
El Cantro	1940	6.6	Several dikes
Hebgen Lake	1959	7.6	1 dam
Alaska	1964	8.4	1 dam
In Japan			
Kanto	1923	8.2	3 dams
Ojica	1939	6.6	74 dams
Fukui	1948	7.3	1 dam
Kita-Moto	1961	7.0	0
Tokachi-Oki	1963	7.8	93 dams
Izu-Oshima	1978	7.0	1 dam
In China			
Bachu	1961	6.8	
Longyao	1966	6.8	
Ningji and Dongwang	1966	7.2	
Bohai Gulf	1969	6.4	
Tonghai	1970	7.7	112 dams
Haicheng	1975	7.3	
Longlin	1976	7.3	
Tengshan	1976	7.8	
Songpan	1976	7.2	
Liyang	1979	6.0	

**Table 2:** Soil properties of the Upper San Fernando dam.

Layer number	*γ*_dry_ (kN/m^3^)	*γ*_wet_ (kN/m^3^)	*E* (MN/m^2^)	*V*_*S*_ (m/s)	*c* (kN/m^2^)	*φ* (degree)	*ν*	*K* (m/day)
1	20.6	22	376.6	252	5.124	28	0.4	0.06
2	18	19.2	198	200	2	37	0.35	0.06
3	17.9	19.2	197	200	2	37	0.35	0.06
4	18	19.2	340	262	2	37	0.35	0.06
5	17.9	19.2	338	262	2	37	0.35	0.06
6	18	19.2	544	330	2	37	0.35	0.06
7	17.9	19.2	570	340	2	37	0.35	0.06
8	19	20.3	539	315	2	37	0.4	0.06
9	19	20.3	1640	550	2	37	0.4	0.04

**Table 3:** Soil properties of the Kitayama dam.

Layer number	*γ*_dry_ (kN/m^3^)	*γ*_wet_ (kN/m^3^)	*E* (MN/m^2^)	*V*_*S*_ (m/s)	*c* (kN/m^2^)	*φ* (degree)	*ν*	*K* (m/day)
1	19.5	20.60	8.519*E* + 5	405.8	2	46	0.3	0.06
2	19.5	20.60	8.390*E* + 5	401.3	2	48	0.31	0.06
3	19.5	20.60	6.238*E* + 5	347.2	2	45	0.3	0.06
4	19.5	20.60	6.689*E* + 5	359.6	2	48	0.3	0.06
5	19.5	20.60	6.881*E* + 5	364.7	2	46	0.3	0.06
6	19.5	20.60	8.562*E* + 5	406.8	2	46	0.3	0.06
7	19.5	20.60	7.884*E* + 5	386	2	44	0.3	0.09
8	19.5	20.60	5.079*E* + 5	313.3	2	44	0.3	0.09
9	19.5	20.60	2.048*E* + 5	215.7	2	44	0.3	0.09
10	27.5	29.1	3.655*E* + 5	215.7	63.7	—	0.3	86.4
11	27.5	29.1	6.472*E* + 5	297.8	63.7	—	0.3	86.4
12	27.5	29.1	8.792*E* + 5	347.1	22.5	39	0.3	82
13	27.5	29.1	1.201*E* + 5	408.7	22.5	39	0.3	82
14	19.3	20.4	2.385*E* + 5	216.8	63.7	—	0.3	0.06
15	19.2	20.5	2.472*E* + 5	210	63.7	—	0.33	0.06
16	19.3	20.4	4.186*E* + 5	280.6	63.7	—	0.35	0.06
17	19.2	20.5	4.119*E* + 5	273.1	63.7	—	0.36	0.06
18	19.3	20.4	7.105*E* + 5	366.9	63.7	—	0.34	0.06
19	19.2	20.5	6.944*E* + 5	357.1	63.7	—	0.36	0.06
20	19.3	20.4	9.007*E* + 5	408.6	63.7	—	0.37	0.06
21	19.3	20.4	1.023*E* + 6	437.1	63.7	—	0.36	0.06
22	19.2	20.5	9.404*E* + 5	420.1	63.7	—	0.35	0.06
23	21.7	23.64	1.61*E* + 6	521.6	2	30	0.34	0.06
24	23.1	24.53	9.36*E* + 6	1222	2	28	0.33	0.1

**Table 4:** Seismic coefficients for Upper San Fernando and Kitayama dams under earthquake loading.

Layer number	Force $\overset{-}{F}$ (kN)	Height of layer (m)	Weight *W* (kN)	Seismic coefficient $C=\overset{-}{F}/W$
Upper San Fernando dam				
1	74	0.8	247	0.311
2	319.2	2.8	1205.4	0.272
3	675.4	4	3023.2	0.254
4	780.5	2.9	3252	0.241
5	870	2.2	3766	0.236
6	1530	3.14	6486	0.235
7	1712.3	3.3	7354.8	0.232
8	1811.1	3.06	7879.2	0.229
9	1895.3	2.95	8739.5	0.216

Kitayama dam				
1	386	2.9	891.3	0.47
2	497	2.8	1420	0.35
3	877	3.5	3043.5	0.29
4	957	2.8	3385.2	0.27
5	1070	2.5	3742.5	0.27
6	1252	2.33	4891.7	0.25
7	1716	2.76	5765.6	0.25
8	1419	2.11	5234.8	0.26
9	2163	3.62	7846.6	0.23
10	681	0.78	3242.8	0.21
